# Environmentally Friendly Photothermal Membranes for Halite Recovery from Reverse Osmosis Brine via Solar-Driven Membrane Crystallization

**DOI:** 10.3390/membranes14040087

**Published:** 2024-04-10

**Authors:** Marco Aquino, Sergio Santoro, Antonio Politano, Giuseppe D’Andrea, Alessio Siciliano, Salvatore Straface, Mauro Francesco La Russa, Efrem Curcio

**Affiliations:** 1 Department of Environmental Engineering, University of Calabria (DIAm-UNICAL), Via P. Bucci, CUBO 44/A, 87036 Rende, Italy; marco.aquino@unical.it (M.A.); giuseppe.dandrea@unical.it (G.D.); alessio.siciliano@unical.it (A.S.); salvatore.straface@unical.it (S.S.); efrem.curcio@unical.it (E.C.); 2 Department of Physical and Chemical Sciences, University of L’Aquila, Via Vetoio, 67100 L’Aquila, Italy; antonio.politano@univaq.it; 3 Department of Biology, Ecology and Earth Sciences, University of Calabria (DiBEST-UNICAL), Via P. Bucci, CUBO 12/B, 87036 Rende, Italy; mlarussa@unical.it

**Keywords:** photothermal membranes, green solvent, graphene, photothermal membrane crystallization, brine valorization

## Abstract

Modern society and industrial development rely heavily on the availability of freshwater and minerals. Seawater reverse osmosis (SWRO) has been widely adopted for freshwater supply, although many questions have arisen about its environmental sustainability owing to the disposal of hypersaline rejected solutions (brine). This scenario has accelerated significant developments towards the hybridization of SWRO with membrane distillation–crystallization (MD-MCr), which can extract water and minerals from spent brine. Nevertheless, the substantial specific energy consumption associated with MD-MCr remains a significant limitation. In this work, energy harvesting was secured from renewables by hotspots embodied in the membranes, implementing the revolutionary approach of brine mining via photothermal membrane crystallization (PhMCr). This method employs self-heating nanostructured interfaces under solar radiation to enhance water evaporation, creating a carefully controlled supersaturated environment responsible for the extraction of minerals. Photothermal mixed matrix photothermal membranes (MMMs) were developed by incorporating graphene oxide (GO) or carbon black (CB) into polyvinylidene fluoride (PVDF) solubilized in an eco-friendly solvent (i.e., triethyl phosphate (TEP)). MMMs were prepared using non-solvent-induced phase separation (NIPS). The effect of GO or GB on the morphology of MMMs and the photothermal behavior was examined. Light-to-heat conversion was used in PhMCr experiments to facilitate the evaporation of water from the SWRO brine to supersaturation, leading to sodium chloride (NaCl) nucleation and crystallization. Overall, the results indicate exciting perspectives of PhMCr in brine valorization for a sustainable desalination industry.

## 1. Introduction

The water crisis is an increasing global plague that could affect more than three billion people by 2050 [1]. While the demand for water is expected to grow consistently with the global population and industrialization [2,3], its accessibility is compromised by climate change and pollution [4]. This scenario has promoted the boom of desalination technologies implemented on every continent to produce high-quality freshwater from seawater or brackish water [5]. According to the International Desalination and Reuse Association (IDRA), more than 22,750 plants currently produce more than 110 × 10^6^ m^3^ of freshwater every day [6]. Seawater reverse osmosis (SWRO), a membrane-based technology, is the most important desalination process, requiring just 3–6 kWh·m^−3^ of energy, which is lower than the 10–16 kWh·m^−3^ required in thermal operations [7,8]. On the other hand, SWRO operates at a recovery factor of ca. 50% imposed by osmotic and concentration polarization phenomena, leading to the production of a massive volume of hypersaline byproducts (i.e., brine). Although brine is usually disposed of in the sea, its high salinity adversely impacts marine and coastal ecosystems, causing eutrophication [9]. Consequently, several studies have focused on the implementation of novel membrane-based strategies for the post-treatment of brine, aiming at improving the water recovery factor [10,11], harvesting blue energy [12,13], and extracting dissolved minerals [14], embracing the principles of process intensification, the circular economy, and zero-liquid discharge [15].

Interestingly, membrane distillation–membrane crystallization (MD-MCr) has the potential to meet the above-mentioned requirements, opening the door for a circular blue economy in the desalination sector [16,17]. The nucleus of an MD-MCr process is a hydrophobic microporous membrane that allows for the permeation of water vapor and rejection of the liquid aqueous phase with dissolved salts [18,19]. MD-MCr guarantees a high-water recovery factor from brine up to its supersaturation and the consequent extraction of dissolved salts [20]. The economic feasibility of MD-MCr on a real scale relies heavily on energy consumption [21,22] because it is a hybrid membrane–thermal process with pronounced heat losses [23,24]. In fact, the sum of the latent heat consumed for the vaporization and condensation of the vapor and the heat conducted through the membrane provoke the emergence of temperature polarization (TP) with a consequent improvement in the specific energy consumption in the order of 100 kWh·m^−3^ [25,26].

In recent decades, advancements in hybridization approaches, such as combining MD-MCr with waste heat or renewable sources, have been proposed for sustainable heat harvesting to compensate for TP [27,28]. Interestingly, the recent embodiment of nanometric hotspots into hydrophobic polymers has ensured the development of self-heating photothermal membranes capable of harvesting heat at the boundary layer of the feed solution, thus reversing the TP [29].

Beyond the pioneering concept of photothermal MD (PhMD) for desalination operation [30,31], our recent studies demonstrated the opportunity to exploit photothermal membranes to dehydrate brine up to their supersaturation, inducing the crystallization of dissolved salts [29,32]. This operation, called photothermal MCr (PhMCr), can have a considerable impact on the practical feasibility of the revolutionary concept of seawater and brine mining [33,34]. 

For example, NiSe and CoSe photothermal nanoparticles have been employed for the synthesis of composite membranes, enabling the rapid solar-driven dehydration of brine [35] and triggering the consequent heterogeneous nucleation and crystallization of NaCl [36]. Among the wide variety of photothermal nanometric hotspots, 2D materials present the relevant advantage of a high surface-area-to-volume ratio, which enhances the light absorption and consequently the photothermal efficiency [37]. This was proved in our case studies on PhMCr carried out with a mixed matrix membrane (MMM) loaded with photothermal semiconductors (i.e., tungsten disulfide (WS_2_)) employed for lithium extraction [38] or vertically aligned graphene membranes for the recovery of inorganic salts from brine [39]. In the latter case, membranes were prepared via sophisticated synthetic routes (i.e., chemical vapor deposition), limiting their practical application on a large scale. Nevertheless, viable strategies (i.e., phase inversion, coating, and electrospinning) have already been employed for the embodiment of graphene into/onto polymeric membranes utilized in MD operations, with the benefit of providing photothermal, photocatalytic, and antibacterial behaviors [40,41,42]. 

In this study, photothermal MMMs were prepared by the embodiment of graphene oxide (GO) at different loadings in polyvinylidene fluoride (PVDF) membranes prepared via non-solvent-induced phase separation (NIPS), an easy and scalable synthetic route. Triethyl phosphate (TEP), chosen as a green solvent, was utilized to solubilize the polymer, providing an environmentally friendly alternative to the conventional solvents used in the NIPS process [43]. Notably, common solvents in the membrane industry, such as N,N-dimethylformamide (DMF), N,N-dimethylacetamide (DMAc), and N-methyl-2-pyrrolidone (NMP) [44], are classified as extremely hazardous and toxic according to the Registration, Evaluation, Authorization, and Restriction of Chemicals (REACH) Regulation of the European Union Chemical Agency. Consequently, substitution of these solvents is necessary due to their detrimental impacts on the environment and human health [45]. TEP offers a more sustainable solution to this problem because it is nontoxic and readily biodegradable, thus reducing wastewater pollution and human health risks, and does not present problems of bioaccumulation or persistence in the environment [46]. 

To highlight the advantages of using 2D GO flakes, PVDF membranes were also doped with carbon black (CB) for comparison. Characterizations were focused on examining the effect of incorporating carbon-based hotspots on both the morphological properties and photothermal behavior of MMMs. The photothermal effect was utilized in PhMCr experiments to boost the vaporization of water from the SWRO brine up to the generation of supersaturation conditions responsible for the nucleation and crystallization of sodium chloride (NaCl). Overall, this study emphasizes the benefits of the embodiment of photothermal 2D materials in MD and MCr membranes for desalination and brine valorization.

## 2. Experimental Section

### 2.1. Materials

Polyvinylidene fluoride (PVDF, grade 6010) was supplied by Solvay Specialty Polymers (Bollate, Italy). Triethyl phosphate (TEP) was purchased from Sigma Aldrich (Milan, Italy) and was used for PVDF solubilization without further purification. Polyvinylpyrrolidone (PVP, grade K17) purchased from BASF (Ludwigshafen, Germany) and polyethylene glycol (PEG, grade 200) purchased from Sigma Aldrich (Milan, Italy) were used as the pore-forming additives. Graphene oxide (GO) powder made of 15–20 sheets that were 4–10% edge-oxidized with a density of 1.8 g·cm^−3^ was purchased by Sigma-Aldrich (Milan, Italy). Activated charcoal, also referred to as carbon black (CB), powder with a density of 2.31 g·cm^−3^ was purchased from Merck KGaA (Darmstadt, Germany).

PhMCr experiments were performed using synthetic SWRO brine containing Na^+^, Mg^2+^, K^+^, and Ca^2+^ as the major cations and Cl^−^, SO_4_^2−^, CO_3_^−^, and NO_3_^−^ as the major ions (Table 1). Before conducting the PhMCr tests, the brine was treated with sodium bicarbonate (NaHCO_3_) to achieve a CO_3_^2−^/Ca molar ratio of 1.3. This procedure is essential to reduce the risk of inorganic scaling, which is primarily caused by calcium-based compounds. The addition of NaHCO_3_ proved to be highly effective in selectively removing Ca^2+^ ions from SWRO, causing them to precipitate as CaCO_3_ or CaSO_4_. This was recently confirmed by Molinari et al. [47]. The reagents for synthesizing the artificial SWRO brine were purchased from Carlo Erba Reagents s.r.l. (Milan, Italy).

### 2.2. Membrane Preparation

**Figure 1:**
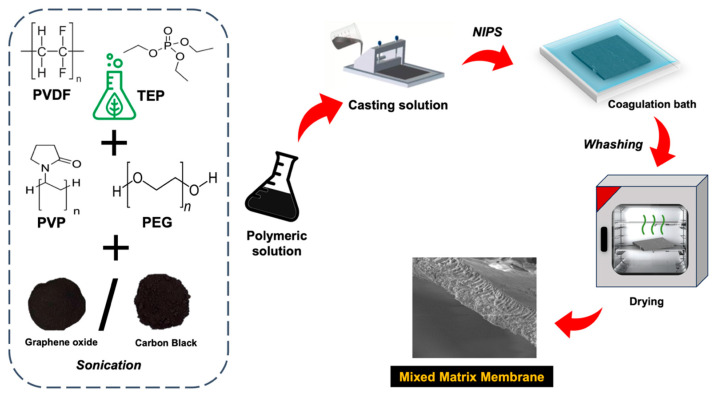
Preparation of green photothermal MMMs based on the embodiment of GO or CB in PVDF.

Photothermal MMMs were fabricated according to the NIPS technique schematized in Figure 1. To obtain a homogeneous dispersion of carbon-based NPs, GO or CB powder was preliminarily dispersed into TEP via sonication (Sonica2200ETH, Soltec, Milan, Italy) for 30 min. Subsequently, the polymers (PVDF, PEG, and PVP) were added to the dispersion of CB or GO and solubilized overnight by magnetic stirring (100 rpm and 70 °C). The concentration of the photothermal filler with respect to the PVDF was 0 wt.%, 2.5 wt.%, 5 wt.%, and 10 wt.%. The PVDF, PEG, and PVP concentrations in the doped solutions were fixed at 15 wt.%, 20 wt.%, and 5 wt.%, respectively. The compositions of the polymeric solutions used for the preparation of photothermal MMMs are listed in Table 2.

Before the casting procedure, the homogeneous PVDF solutions were degassed for 2 h at 70 °C to ensure the removal of the entrapped air. Photothermal MMMs were prepared by casting the polymeric solutions with a doctor blade (Elcometer, Manchester, UK) set at 0.30 mm of thickness. Nascent MMMs were immersed in a mixture of water and ethanol (50/50 wt.%/wt.%) for 3 h (Figure 1). The as-formed MMMs were kept overnight in distilled water at room temperature and then washed in distilled water at 60 °C for 2 h (every 30 min, the water of the washing bath was changed) to remove any traces of solvent and additives (i.e., PEG and PVP). Finally, the membranes were dried for 8 h at 50 °C.

### 2.3. Membrane Characterization

The morphologies of the membranes were observed using scanning electron microscopy (SEM, EVO MA10, Zeiss, Oberkochen, Germany) at a beam energy of 10 kV. The samples were fixed on stubs using conductive carbon tape and sputter-coated with a thin graphite film before the analysis. Both membrane surfaces were examined. For cross-sectional observations, the membranes were frozen by immersion in liquid nitrogen (1 min) and subsequently fractured to preserve their microporous structure. 

A setup for contact angle (θ) measurements (Drop Shape Analyzer-DSA30, Kruss GmbH, Hamburg, Germany) was employed to evaluate membrane hydrophobicity according to the sessile drop method at ambient temperature.

Pore size and bubble point measurements were conducted using a capillary flow porometer (INNOVA-500NX, Porous Materials Inc., Ithaca, NY, USA) under the management of the software Capwin (https://pmiapp.com/, accessed on 8 April 2024, Porous Materials Inc., Ithaca, NY, USA). The wet-up/dry-up method was employed using Galwet^®^ (surface tension of 15.9 dyne·cm^−1^) as the wetting liquid. The results were processed using Caprep software (https://pmiapp.com/, accessed on 8 April 2024, Porous Materials Inc., Ithaca, NY, USA).

The porosities (*ε*) of the membranes were determined using a gravimetric method. Briefly, the membrane was weighed before (dry) and after immersion in kerosene for 24 h (wet). The value of ε was calculated using the following equation:

(1) $$\varepsilon \;\left(\%\right)=\left\{\frac{(W_{w}-W_{d})/\rho_{k}}{(W_{w}-{\;W}_{d})/\rho_{k}+\frac{{\;W}_{d}}{\rho_{m}}}\right\}∙100$$

where *W_W_* is the weight of the wet membrane, *W_d_* is the weight of the dry membrane, ρ*_k_* is the kerosene density (0.81 g⋅cm^−3^), and ρ*_m_* is the density of the membrane material estimated from the PVDF (1.8 g⋅cm^−3^) [48], GO (1.9 g⋅cm^−3^) [49] and CB (2.3 g⋅cm^−3^) [50] densities. Three measurements were performed for each membrane to calculate the average values and standard deviations of the porosities. 

Ultraviolet and visible (UV-Vis) absorption was measured using a spectrophotometer (Shimadzu UV-1601, Kyoto, Japan). The photothermal response of the MMMs was measured using an infrared (IR) camera (FLIR, model T660, Wilsonville, OR, USA) with a sensitivity of ca. 0.02 °C and a resolution of 640 × 480 pixels. 

X-ray diffraction (XRD) analysis was performed on crystallized salts with a D8 Advance Bruker Coatings diffractometer (Billerica, MA, USA) with Cu Kα radiation (operating conditions: 2θ step size of 0.02°at 2 s/step, analytical range from 3° to 65°) to identify the constituent mineralogical phases of the obtained crystals. 

### 2.4. Photothermal Membrane Crystallization Experiments

Solar-driven PhMCr experiments were conducted using distilled water or SWRO brine with a micro-peristaltic pump (Reglo, Ismatec, USA) at a flow rate of 10 mL·min^−1^. The membrane (active area 3.14 cm^2^) was positioned in a module of polylactic acid (PLA) designed with Autodesk 123D^®^, printed using a 3D printer (model Tornado, TEVO, China), and equipped with a transparent window exposing the feed surface to external radiation. During the PhMCr test, the membrane module was irradiated by light generated from a solar simulator (Abet Technologies, Milford, CT, USA) mimicking the sunlight spectrum with an irradiance of 1 sun (P_in_ = 1000 W⋅m^−2^) over an area field of 35 mm in diameter. 

The evaporation rate was periodically determined by weighing the feed with an analytical balance (Gilbertini, Model E 50 S/3, Novate Milanese, Italy). The temperature during the experiments was 25.8 ± 0.5 °C, and the relative humidity was maintained at 47 ± 4%. Experiments with solar-driven PhMCr were repeated three times, and the mean values of the water evaporation fluxes and experimental errors were then calculated. 

After the experiments, the obtained crystals were collected and examined under a Nikon industrial microscope (Eclipse LV100ND; Nikon, Tokyo, Japan). The cumulative function *F*(*L*) for the crystal size distribution was determined (population > 100 crystals), and the coefficient of variation (*CV*) was calculated as follows:

(2) $$\;\;CV=\frac{L84\%-L16\%}{2\;L50\%}$$

where the crystal size (*L*) was obtained from the *F*(*L*) curve at a specified percentage (16, 50, or 84%) [51].

## 3. Results and Discussion

### 3.1. Membrane Properties

The thickness of the MMMs ranged from 150 to 166 μm for the membrane doped with CB, while the thickness of the GO membranes presented a thickness range from 133 to 150 μm. These values were similar to the thickness observed for the blank PVDF, which was 161 ± 3 μm. 

**Figure 2:**
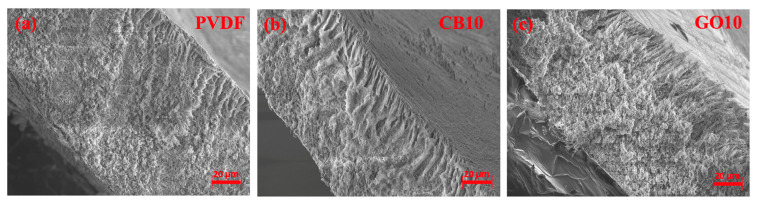
SEM images (magnification: 1000×) of (**a**) blank PVDF membrane (PVDF); (**b**) MMM loaded with 10 wt.% of CB (CB10); and (**c**) MMM loaded with 10 wt.% of GO (GO10).

SEM inspections of the membrane cross-sections revealed the poor impact of both photothermal nanomaterials on the morphology of MMMs in comparison with the blank PVDF membrane (Figure 2). In all cases, the membranes demonstrated a heterogeneous microporous structure because the surface in contact with the coagulation bath during the NIPS stage (top surface) presented a finger-like morphology supported by a sponge-like layer (bottom surface). This is because of the differences in the kinetic and thermodynamic aspects governing NIPS between the two surfaces of the nascent membranes [52], as already discussed by Marino et al. for PVDF membranes prepared using TEP as the solvent [53]. The top surface was obtained by direct exposure of the cast polymeric solution to the coagulation bath, encouraging rapid demixing and the consequent formation of finger-like pores [54]. Upon diffusion, the non-solvent induced a delayed NIPS of the polymeric solution on the bottom surface with a consequent sponge-like morphology [55]. In addition, Pagliero et al. observed a sponge-like morphology for PVDF membranes prepared using TEP as the solvent, doped with GO or CB, and employing ethanol as the non-solvent [56]. In our case, the presence of water in the coagulation bath accelerated the NIPS stage, leading to the appearance of finger-like pores [57,58]. It is worth mentioning that the presence of finger-like pores in MD-MCr membranes is highly desirable because they offer minimal resistance to the mass transport of water vapor and reduce membrane conductivity.

The different surface morphologies affected their hydrophobicity; the bottom spherulitic-like surfaces presented higher roughness, increasing the contact angle according to the Wenznel equation [12]. In fact, the bottom surface of the GO10 membrane showed a contact angle of ~119°, 20° higher than that of the smooth finger-like top surface (Table 3). Furthermore, despite the inherently hydrophilic nature of the photothermal fillers, their inclusion resulted in an improvement in surface hydrophobicity. This phenomenon can be attributed to the enhanced surface roughness induced by the presence of carbon-based hotspots. For instance, Pagliero et al. observed a contact angle above 140° for PVDF membranes loaded with 7.5 wt.% of GO or CB [56]. Additionally, Mortaheb et al. observed an improvement in the mean roughness of PVDF membranes from 34 nm to 136 nm as a consequence of the embodiment of 1 wt.% of 2D flakes [59].

Despite the poor impact on membrane morphology, the organization of the polymeric chains during NIPS was disturbed by the presence of photothermal nanoparticles, leading to an increase in porosity. In fact, the blank PVDF membranes showed a porosity of 70.6%, which was lower than the values measured for the developed MMMs, with the exception of CB 10 (Table 3). In fact, the porosity of the PVDF-based membranes progressively increased to 78.1% and 82.6%, raising the CB concentration to 2.5 wt.% and 5 wt.%, respectively. A further improvement in the CB content to 10 wt.% decreased the porosity to 67.0% because of the high content of fillers decreasing the void space in the PVDF matrices. This effect was not observed for the MMMs prepared with GO with a porosity of 85.2% for a loading of 10 wt.% thanks to the effective dispersity of the 2D flakes [56,60]. In all cases, MMMs presented sub-micrometric pore sizes with average values in the order of 0.1–0.2 μm (Table 3). These results are in accordance with the recommended pore size for MD-MCr applications, finding a balance between the need to maximize mass transport and to minimize the risk of pore wetting [61]. In fact, the average pore size for the blank PVDF was 0.092 μm, with the largest pore detected at 0.219 μm (bubble point of 2.08 bar). The variation in the concentration of CB nanoparticles weakly influenced the pore size because fluctuations below 25% were observed in the average pore size. On the other hand, the loading of 2.5 wt.% of GO in PVDF led to an increase in the average pore size by 2-fold (from 0.092 μm to 0.184 μm), coherent with the increase in porosity (from 70.6% to 84.6%). The further improvement in the GO concentration provoked a gradual reduction in the average pore size down to 0.075 μm for the embodiment of the 2D flakes of 10 wt.%.

**Figure 3:**
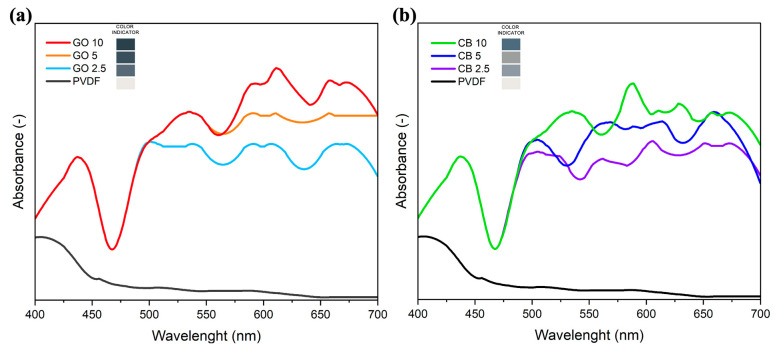
The visible spectra of the membranes with different GO (**a**) and CB (**b**) loading.

The color of the MMMs changed from white to gray to black as the loading of GO or CB nanofillers increased. This change in color reflects the enhanced absorption in the visible region of the electromagnetic spectrum, as demonstrated by the spectra (Figure 3). In addition, the absorption spectra confirm the poor interaction of the neat PVDF with the irradiation, while the carbon-based nanomaterial exhibits an intense and wide absorption peak in the range of 470–700 nm (Figure 3), matching the peak of sunlight irradiation on Earth.

### 3.2. Photothermal Performance

**Figure 4:**
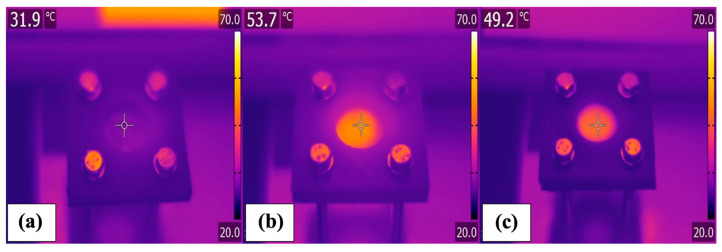
Infrared images of (**a**) blank PVDF, (**b**) GO 10, and (**c**) CB 10 membranes. Temperature measurements were conducted within the designated membrane area indicated by the target symbol.

The GO/CB-PVDF MMMs were exposed to artificial solar irradiation to assess their photothermal efficiencies. The irradiated blank PVDF barely exhibited significant variation from the environment, showing a temperature of 31.2 °C under sunlight irradiation (Figure 4a). On the other hand, the IR camera revealed that the absorbed radiation was efficiently converted into heat by the carbon-based hotspots, leading to an improvement in the membrane temperature (Figure 4b,c). 

**Figure 5:**
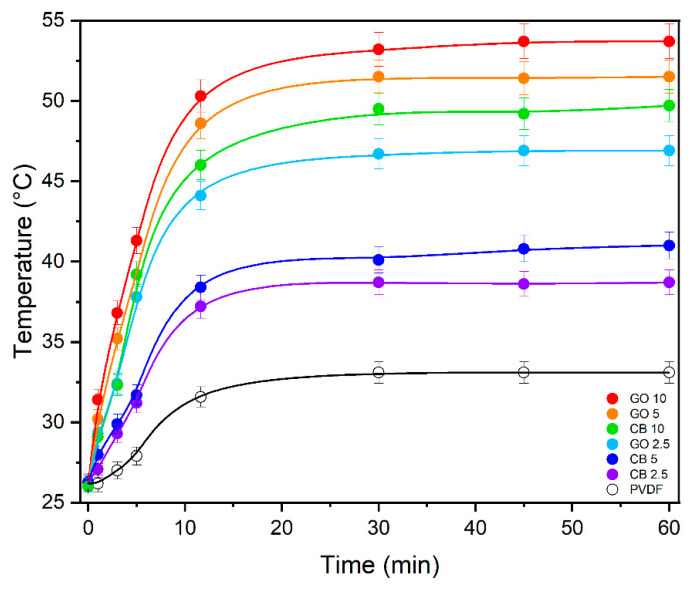
Time evolution of the temperature profile of MMMs and PVDF membrane under solar simulator.

When observing the temperature evolution of the membranes with respect to irradiation time, it was observed that the MMMs quickly reached a steady state (<5 min) (Figure 5). The highest GO loading increased the membrane temperature from 26.1 °C (room temperature) to 53.7 °C. An interesting photothermal behavior was also observed for the GO5 and GO2.5 membranes, achieving temperatures of 49.9 °C and 45.0 °C, respectively. 

The CB-based MMMs exhibited a lower photothermal efficiency, and CB10 guaranteed a membrane temperature of 49.2 °C under the sunlight radiation. Evidently, a decrease in the nano-heater content reduced the photothermal effect in CB 2.5 and CB5, reaching temperatures of 38.4 and 40.6 °C, respectively (Figure 5). 

The difference in the photothermal response between CB and GO is attributed to the distinctive organization of carbon atoms in the nanomaterials. In general, carbon-based materials act as black bodies that efficiently absorb the entire spectrum of sunlight radiation by promoting the excitation of loose electrons from π to π* orbitals. The return of electrons to their ground states is coupled with the transfer of energy to the lattice by phonons, releasing the heat responsible for the photothermal effect. In CB, the thermal energy is significantly altered by phonon scattering at boundaries or by disorder, while GO exhibits outstanding thermal conductivity because heat is ruled by the intrinsic properties of the highly ordered sp^3^ or sp^2^ lattice, leading to an effective light-to-heat conversion [62].

Sunlight-activated nano-heaters radically affect the efficiency of water vaporization, increasing with the temperature of the membranes. The evaporative flux (*J_w_*) is proportional to the difference in feed water vapor pressure at the membrane surface ($p_{w}^{membrane}$) and the partial pressure of water vapor in air ($p_{w}^{air}$):

(3) $$J_{w}=K\left(p_{w}^{membrane}-p_{w}^{air}\right)$$

This difference in water vapor pressure also consists of the driving force for MD-MCr. The value was estimated for pure water using the following equation:

(4) $$\;\;\left(p_{w}^{membrane}-p_{w}^{air}\right)=\left[p_{w}^{0}\left(T_{f}^{*}\right)-p_{w}^{0}(T_{air})∙\frac{\varphi }{100}\right]$$

where $p_{w}^{0}(T_{f}^{*})$ is the saturated water vapor pressure at the membrane surface temperature (*T_f_*), $p_{w}^{0}(T_{air})$ is the saturated water vapor pressure at the actual dry bulb temperature (*T_air_*), and *φ* is the relative humidity.

The improvement in the value of $T_{f}^{*}$ promoted by the photothermal behavior boosts the vaporization of the water by raising the value of $p_{w}^{0}$ exponentially, as confirmed by the Clausius–Clapeyron relation. For instance, while at room temperature, the value of $p_{w}^{0}$ was solely 0.033 atm; the solar-driven self-heating effect enabled hitting a $p_{w}^{0}$ of 0.145 atm, resulting in facilitated vaporization. In fact, the evaporation rates ranged from 0.560 L·m^−2^·h^−1^ for pristine PVDF to 0.904 L·m^−2^·h^−1^ for the membrane loaded with 10 wt.% GO (GO10). In the case of CB10, the evaporation rate was 0.847 L·m^−2^·h^−1^, 6.30% lower than that of GO10. The presence of photothermal particles in the membrane impacted K since the bare PVDF membrane and its modification by the inclusion of 10 wt.% CB and GO exhibited values of 31.5, 8.39, and 7.08 L·m^−2^·h^−1^·atm^−1^, respectively. 

**Figure 6:**
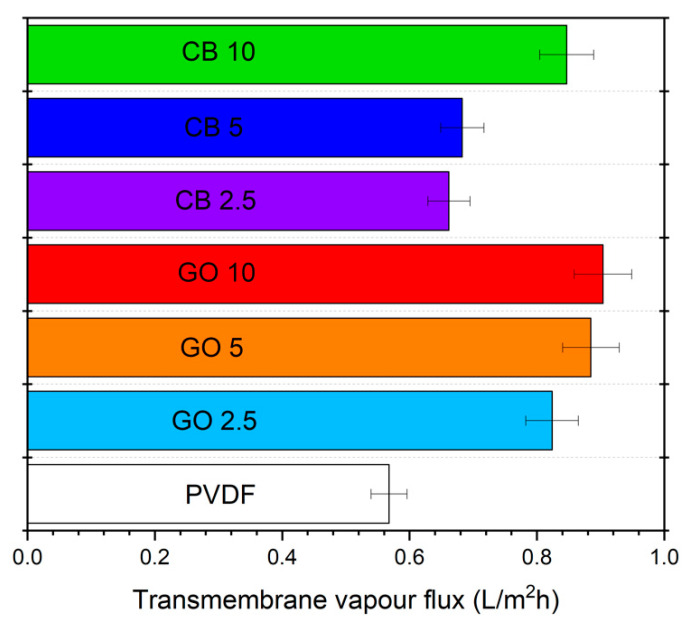
Evaporation rate of distilled water using PVDF membranes and GO or CB MMMs under solar radiation.

As expected, the gradual reduction in the photothermal effect with the concentration of carbon-based nanomaterials matched with a decrease in the photothermal efficiency (*η_v_*) calculated as:

(5) $$\;\;\;\;\;\eta_{v}=\frac{J_{w}{∙\lambda }_{v}}{P_{in}};$$

where *λ_v_* is the latent heat of vaporization of the water. Under solar irradiation, *η_v_* significantly increased from 30% for the pure PVDF to 65% for the PVDF loaded with the highest concentration of CB nanofiller (CB10), and to 72% for GO10 (Figure 6).

### 3.3. Photothermal Membrane Crystallization

After confirming the effectiveness of the photothermal effects with distilled water, MMMs were tested in sunlight-driven PhMCr to extract salts and recover water from the SWRO brine. 

**Figure 7:**
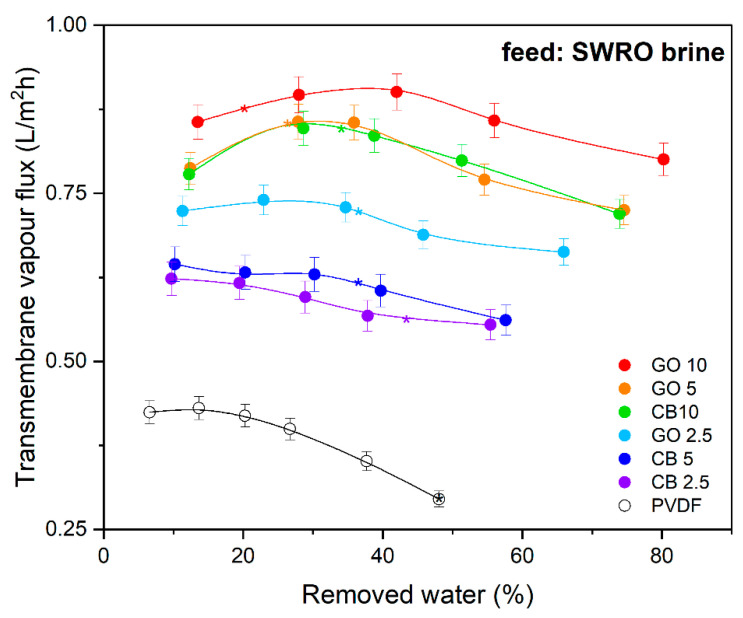
Evaporation rate from artificial SWRO brine in PhMCr under solar radiation (* nucleation).

Figure 7 shows the evaporation rate from the SWRO brine as a function of the recovered water (%), evidencing that the higher temperature of the photothermal membranes boosted the vaporization of the water. A higher *J_w_* value was observed for the MMM loaded with 10 wt.% of GO (0.910 L·m^−2^·h^−1^), more than 2-fold higher than the one observed for blank PVDF (0.446 L·m^−2^·h^−1^) (see Figure 7). This latter value is comparable to the evaporation rate from brine observed in the absence of a membrane (0.403 L·m^−2^·h^−1^), highlighting the irrelevant impact of the PVDF membrane on *J_w_*. As expected, the reduction in the GO concentration from 10 wt.% to 2.5 wt.% led to a reduction in the photothermal behavior, with a consequent lowering of *J_w_* from 0.910 L·m^−2^·h^−1^ to 0.740 L·m^−2^·h^−1^. The lower photothermal efficiency of CB compared to that of GO led to an inferior *J_w_* from the brine: the CB10 membranes presented a maximum evaporation rate of 0.854 L·m^−2^·h^−1^, close to the value observed for GO2.5 (0.740 L·m^−2^·h^−1^). 

In all cases, the flux slightly increased in the early stages of the experiments because of the time required to achieve the steady state of the photothermal membrane–SWRO brine system. Subsequently, *J_w_* progressively decreased during the PhMCr experiments because of the gradual improvement in the concentration of the solutes by evaporating the recovered water. In fact, the partial pressure of the water in a solution ($p_{w}(T_{f}^{*})$) depends on its molar fraction ($x_{w}$) and activity coefficient ($\gamma_{w}$), as described by the following equation:

(6) $$\;\;p_{w}(T_{f}^{*})=p_{w}^{0}\left(T_{f}^{*}\right)∙x_{w}∙\gamma_{w}$$

Thus, the gradual dehydration of the brine led to a coherent decrease in the driving force due to a reduction in both $x_{w}$ (from 0.98 to 0.91) and $\gamma_{w}$ (from 0.97 to 0.74). 

The advantages of using a membrane with higher photothermal efficiency were observed in the reduction in the time required to induce the supersaturation of the brine and, as a consequence, the nucleation of the crystals. The crystals first nucleated on the surface of GO10 because of the rapid dehydration of the brine. With respect to GO10, the nucleation time extended by 34% and 57% for GO5 and CB10, respectively, because of the lower transmembrane vapor fluxes. However, the poor evaporation rate from the bare PVDF membranes delayed the time required for the appearance of crystal nuclei by more than 4-fold.

**Figure 8:**
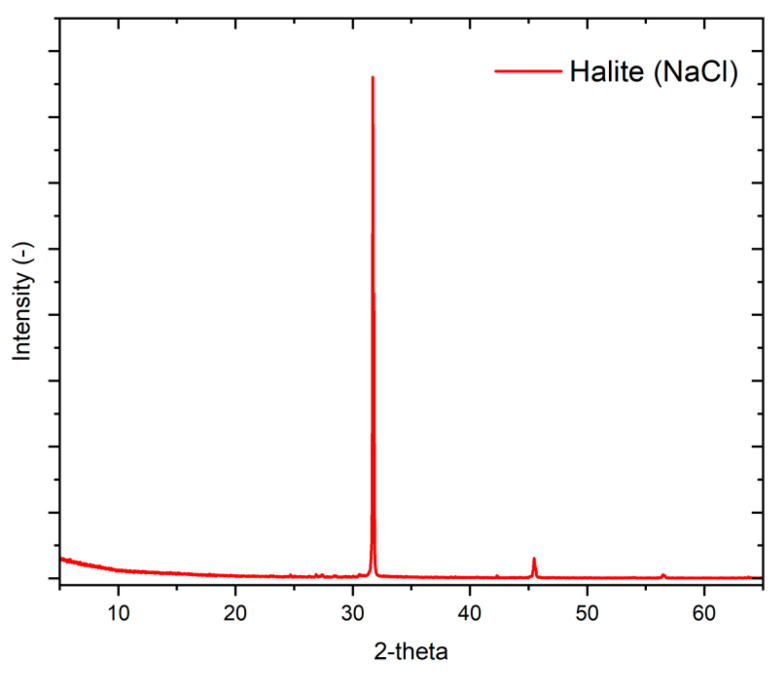
XRD pattern of crystals extracted from artificial SWRO brine in PhMCr experiment performed with GO10.

Post hoc inspection with an optical microscope of the membrane surface employed in the PhMCr experiments revealed the presence of crystals with a cubic structure, which is the characteristic morphology of NaCl. In accordance with the guidelines established by the Joint Committee on Powder Diffraction Standards (JCPDS), the crystal form was confirmed to be halite NaCl by the diffraction peaks at 27.4°, 31.7°, 45.4°, and 56.4°. Thus, the resulting XRD pattern (Figure 8) shows the dominant presence of halite peaks (100% of the total) [63].

**Figure 9:**
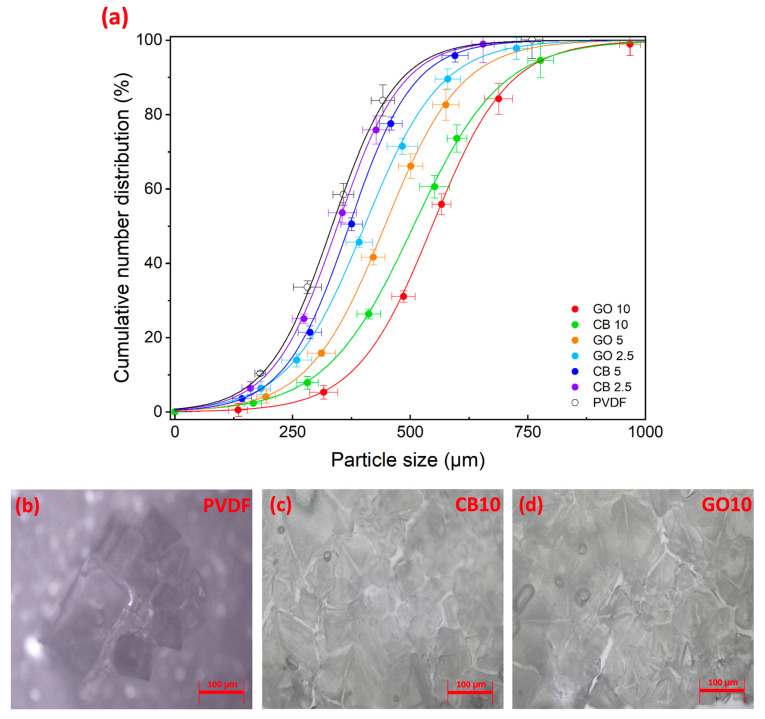
(**a**) Cumulative number distribution of crystals collected on the surface of membranes after PhMCr; microscopic images of crystals extracted on the surface of (**b**) blank PVDF, (**c**) CB10, and (**d**) GO10 after the experiments with PhMCr.

A wide variation in the crystal size distribution was observed for the crystals nucleated on bare PVDF membranes, as evidenced by the large coefficient of variation calculated using the cumulative number distribution function, which reached 250% (Figure 9). This occurred when water slowly evaporated from the solution, favoring the formation of a few initial crystals. Their slow growth combined with the heterogeneity of supersaturated environments leads to the appearance of crystals with widely varying sizes, which is inadequate for industrial applications.

In contrast, the early onset of nucleation, promoted by the photothermal effect of CB and GO, provided nascent crystals with more time to grow in a well-controlled supersaturated environment. Larger crystals were detected on the surfaces of the photothermal MMMs with narrow distributions (Figure 9). Specifically, the average sizes of the final crystalline products were 497 and 583 μm for CB10 and GO10, respectively. In addition, the coefficients of variation of the crystallized salts on the CB-doped MMM tended to be larger than those observed for GO-based membranes. In fact, the crystal growth on GO10 showed a lower dispersion in the size distribution around the mean value (CV = 60% for 10 GO, CV = 74% for 10 CB). This was determined by the better dispersion of GO flakes into PVDF, resulting in a homogeneous membrane surface temperature profile that ensured standardized conditions for nucleation.

Likewise, the population density of crystals on GO10 (1862.02 crystals·cm^−2^) was 3-fold higher than that on the bare PVDF and about +20% higher with respect to the observations on the GO2.5 and CB2.5 membranes. This agrees with classical nucleation theory [38,64]: the boosted vaporization of water on GO10 facilitated nucleation, with the consequent formation of a large number of small nuclei. Furthermore, the shorter nucleation time guaranteed to the nuclei a longer resilience time in a supersaturated solution, promoting their growth. Thus, a high concentration of GO assisted both the nucleation and growth phases, facilitating the extraction of minerals from the SWRO brine by providing a homogeneous supersaturated environment. In fact, the ultimate result was that the GO10 and CB10 membranes achieved crystallization yields of 22–24%, which were more than 7% higher than that of the blank PVDF.

In general, the process of crystallization involves two different stages: the initial nucleation phase followed by the subsequent growth of crystals. In batch crystallization, commonly known as homogeneous crystallization, these stages are indistinguishable and governed by the random movement of solutes [65,66]. A membrane can reduce the energy required for nucleation (referred to as the Gibbs energy barrier, ΔG*) by providing reversible chemical interactions on its surface and creating favorable supersaturated microenvironments on its pore mouths [67,68]. Consequently, the crystals initiate nucleation at an early stage on the membrane surface (i.e., heterogeneous nucleation) [69]. 

The membrane’s contribution to facilitating the crystallization of dissolved salts can be quantified by comparing the ΔG* for heterogeneous nucleation at the membrane interface ($∆G_{het}^{*}$) to that for homogeneous nucleation within the bulk of the solution ($∆G_{hom}^{*}$), as follows [36]:

(7) $$\;\;\;\frac{∆G_{het}^{*}}{∆G_{hom}^{*}}=\frac{1}{4}\left(2+cos\theta \right){\left(1-cos\theta \right)}^{2}{\left[1-\epsilon \frac{{\left(1+cos\theta \right)}^{2}}{{\left(1-cos\theta \right)}^{2}}\right]}^{3}$$

In all the cases, the ratio $\frac{∆G_{het}^{*}}{∆G_{hom}^{*}}$ for the PVDF membrane and the MMMs doped with GO or CB hotspots was below 0.64, underscoring the significant role of the membranes in the heterogeneous crystallization of halite.

However, heterogeneous crystallization leads to a significant deposition of crystallized salts on membrane surfaces (Figure 9), which impedes vapor transport and results in detrimental effects. These effects include a decrease in hydrophobicity, posing a risk of membrane wetting [70] as well as the deterioration of the chemical and mechanical stability [71]. These aspects impose the need for further studies to avoid this phenomenon, focusing on process optimization [72]. 

In fact, this work demonstrated the potentialities of carbon-based MMMs in exploiting solar radiation to boost water vaporization from SWRO brine, resulting in facilitated halite crystallization. Practical applications require a recirculation of the feed at high shear rates to promote the detachment of crystal nuclei from the membrane surface [73]. Beyond the careful control of the hydrodynamic conditions at the membrane surface [74], introducing pulse flow [75], ultrasonication [76], air bubbles [77], or turbulence [78] can effectively prevent the formation of scaling on the membrane surface. Finally, the recovery of crystallized salt from the mother liquor mitigates the impact of fouling/scaling, further advancing the maturity of MCr technology towards continuous operation [79]. Considering these findings and challenges, future research endeavors will delve deeper into optimizing PhMD-MCr processes, exploring different strategies to overcome scaling issues and enhance the efficiency and applicability of this promising technology.

## 4. Conclusions

This study investigates the preparation of green photothermal MMMs by the embodiment of carbon-based nanomaterials exploited in solar-driven PhMCr experiments to boost the vaporization of water from the brine up to its supersaturation and the consequent crystallization of dissolved minerals.

MMMs were prepared via NIPS by preliminary dispersion of the nanoparticles into an environmentally friendly solvent (i.e., TEP), where the polymer (i.e., PVDF) was subsequently solubilized together with the pore-forming agents (i.e., PVP and PEG). In all cases, the membranes exhibited asymmetric structures based on a finger-like layer on a microporous spherulitic structure. The developed MMMs based on the embodiment of GO met the requirements for MD and MCr processes since they presented a porosity above 80%, pore size in the order of 0.07–0.18 μm, and contact angle superior to 96°. On the other hand, the poor dispersity of CB negatively impacted the porosity, falling to 68% for CB10. 

The solar radiation absorbed by the nanoparticles was effectively converted into heat: GO10 reached a surface temperature of 53.7 °C under one sun, while CB10 was heated to 49.2 °C. In the case of the blank PVDF membrane, the temperature under sunlight radiation was just 31.2 °C. As a result, the vaporization of the water increased with the temperature of the membrane surface, and the GO10 MMM secured an evaporation rate from SWRO brine of 0.910 L·m^−2^·h^−1^, 2-fold higher than that of neat PVDF. 

The impact of GO on PhMCr is in line with the photothermal effects, as the surface high evaporative flux facilitated the generation of supersaturation conditions. Therefore, GO10 outperformed the other membranes in terms of the short nucleation time for NaCl from the multi-ionic brine. This offered the nucleated salts a longer resilience time in a supersaturated environment, which promoted their growth. Thus, the superior photothermal efficiency of GO10 resulted in a higher population density (1862.02 crystals/cm^2^) of large crystals (583 μm). Interestingly, the facilitated dispersion of the 2D flakes enabled the generation of homogeneous supersaturation conditions, resulting in crystals with narrow pore sizes. Undoubtedly, the GO10 MMM demonstrated superior performance in PhMCr practice due to its higher light-to-heat photothermal conversion efficiency, resulting in (i) an enhanced vaporization of water, (ii) reduced nucleation time, and (iii) increased mineral recovery.

Our study highlights the development of photothermal membranes for PhMCr application, alongside the acknowledgment of the necessity to evaluate fouling/scaling phenomena. Future research will entail systematic investigations on a larger scale to optimize practical applications, assess the long-term stability, and address membrane fouling/scaling behavior.

Overall, this work demonstrates the benefits of exploiting 2D materials in the development of photothermal membranes that can be exploited in the implementation of circular approaches in desalination via solar-driven PhMCr.

## Figures and Tables

**Table 1:** The ionic composition of SWRO brine measured by ion chromatography.

Ion	Na^+^	Mg^2+^	K^+^	Ca^2+^	Cl^−^	SO_4_^2−^	CO_3_^2−^	NO_3_^−^
Concentration (ppm)	35,902	4236	1356	980	41,642	4398	320	130

**Table 2:** Polymeric solutions used for the preparation of photothermal MMMs.

Membrane	PVDF	GO/CB 2.5	GO/CB 5	GO/CB 10
TEP (wt.%)	60	59.625	59.25	58.5
PVDF (wt.%)	15	15	15	15
PEG (wt.%)	20	20	20	20
PVP (wt.%)	5	5	5	5
NPs: GO/CB (wt.%)	-	0.375	0.75	1.5

**Table 3:** Comparison of thickness, mean pore diameter, pore distribution, and contact angle of the developed photothermal MMMs.

Membrane	Thickness (μm)	Mean Pore (μm)	Porosity (%)	Contact Angle Bottom (°)	Contact Angle Top (°)
Bare PVDF	161 ± 3	0.092 ± 0.007	70.6 ± 0.8	97 ± 2	92 ± 2
CB 2.5	152 ± 1	0.112 ± 0.004	78.1 ± 0.6	96 ± 2	93 ± 2
CB 5	166 ± 2	0.092 ± 0.006	82.6 ± 1.0	110 ± 1	92 ± 1
CB 10	150 ± 3	0.071 ± 0.005	67.0 ± 0.6	113 ± 2	93 ± 2
GO 2.5	150 ± 2	0.184 ± 0.009	84.6 ± 0.7	96 ± 2	94 ± 2
GO 5	136 ± 1	0.126 ± 0.008	80.6 ± 0.4	120 ± 2	96 ± 2
GO 10	133 ± 1	0.075 ± 0.006	85.2 ± 0.6	119 ± 2	99 ± 2

## Data Availability

Data are contained within the article.
